# Natural product manoalide promotes EGFR-TKI sensitivity of lung cancer cells by KRAS-ERK pathway and mitochondrial Ca^2+^ overload-induced ferroptosis

**DOI:** 10.3389/fphar.2022.1109822

**Published:** 2023-01-11

**Authors:** Yinyun Ni, Jiaye Liu, Lingyan Zeng, Ying Yang, Lei Liu, Menglin Yao, Li Chai, Lu Zhang, Yi Li, Li Zhang, Weimin Li

**Affiliations:** ^1^ Institute of Respiratory Health, Frontiers Science Center for Disease-Related Molecular Network (NHC Key Laboratory of Transplant Engineering and Immunology), West China Hospital, Sichuan University, Chengdu, Sichuan, China; ^2^ Department of Thyroid and Parathyroid Surgery, West China Hospital, Sichuan University, Chengdu, Sichuan, China; ^3^ Institute of Core facility, West China Hospital, Sichuan University, Chengdu, Sichuan, China; ^4^ Precision Medicine Center, Precision Medicine Key Laboratory of Sichuan Province, West China Hospital, Sichuan University, Chengdu, Sichuan, China

**Keywords:** lung cancer, EGFR-TKI resistance, manoalide (MA), ROS, mitochondrial Ca^2+^, ferroptosis

## Abstract

**Background:** Manoalide (MA), a proven natural inhibitor of PLA2 has anticancer effects, but its potential application and mechanism as an anticancer drug to promote EGFR-TKI sensitivity in lung cancer cells have not been studied.

**Methods:** KRAS-mutated lung cancer cells and organoids, acquired osimertinib-resistant lung cancer cell lines HCC827OR, were used as EGFR-TKI-resistant models. CCK-8, clone formation, apoptosis assays, and calcein-AM staining were performed to investigate the inhibitory effects of MA in lung cancer cells and organoids. The flow cytometry or confocal microscope was used to detect lipid droplets, ROS, lipid peroxidation, mitochondria Ca^2+^, and iron content. The oxygen consumption rate (OCR) and fatty acid oxidation (FAO) were used to estimate the effect of MA on mitochondrial function.

**Results:** MA inhibits the proliferation of KRAS-mutated lung cancer cells and organoids. In addition, MA induces ER stress in a ROS-dependent mechanism. The ROS induced by MA is mainly in mitochondrial and causes lipid peroxidation, thereby inhibiting mitochondrial FAO metabolism and promoting the accumulation of lipid droplets. MA also suppresses the KRAS-ERK pathway through ROS and promotes the sensitivity of KRAS-mutated lung cancer cells and organoids to osimertinib. Furthermore, MA induces ferroptosis by suppressing the NRF2-SLC7A11 axis and mitochondrial Ca^2+^ overload induced-FTH1 pathways to promote the sensitivity of osimertinib-resistant lung cancer cells to osimertinib.

**Conclusions:** MA is a candidate EGFR-TKI sensitizer in KRAS-mutated and osimertinib-resistant lung cancer cells.

## Graphical Abstract

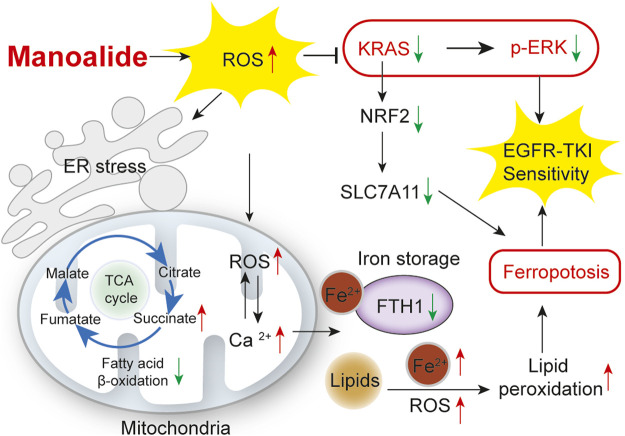

## Introduction

Non-small cell lung cancer (NSCLC) is one of the leading causes of cancer-related deaths and has a poor 5-year survival rate of <15% due to inevitable acquired resistance to antineoplastic drugs, platinum-based chemotherapy, and targeted therapy (Nagasaka and Gadgeel, 2018; Sankar et al., 2020; Siegel et al., 2022). Up to 50% of Asian NSCLC patients harbor EGFR mutations, such as exon 19 deletion (ex19del), the missense mutation in exon 18 (G719X), or exon 21 (L858R) (Goldstraw et al., 2011; Shi et al., 2014; Hirsch et al., 2017). In recent years, the third-generation EGFR-TKI osimertinib has made great progress in the treatment of EGFR-mutated lung cancer (Ramalingam et al., 2018). However, resistance to osimertinib is unavoidable, and KRAS mutations are found in 30% of lung adenocarcinoma patients who cannot benefit from treatment with osimertinib (Thress et al., 2015; Reck et al., 2021). Activation of the RAS-MAPK signaling pathway is a common mechanism of osimertinib resistance in KRAS-mutated lung cancer cells and EGFR-TKI-resistant cells (Zhang et al., 2021). Therefore, in patients with KRAS mutations and EGFR-TKI resistance, there is an urgent need to develop new EGFR-TKI sensitizers and combination strategies to overcome resistance to EGFR-TKIs.

Manoalide (MA) is a marine natural product isolated from sponges in 1980 that has analgesic, anti-inflammatory, and other effects (Silva and Scheuer, 1980; SorienteDe Rosa et al., 1999). The anti-inflammatory activity of MA is due to its inhibition of PLA_2_ (phospholipase A_2_) through irreversible binding to several lysine residues (Folmer et al., 2010). Recent studies have shown that MA also has anticancer effects on oral cancer cells (Wang et al., 2019) and leukemic cancer cells (Lai et al., 2021), but its potential application and mechanism as an anticancer drug have not been widely studied, such as whether it can inhibit lung cancer cells. Since MA reached phase II clinical trials for the treatment of psoriasis and showed a high degree of safety (SorienteDe Rosa et al., 1999). *In vivo* experiments in mice also confirmed the *in vivo* safety of MA (Lai et al., 2021). Therefore, this compound has great potential as an anticancer agent for future development. Cytoplasmic phospholipase A_2_ (cPLA_2_), encoded by the PLA2G4A gene, is the most abundant isoform of PLA_2_ and plays an important role in tumor development (Shimizu et al., 2006). We found through the TCGA database that PLA2G4A is highly expressed in KRAS-mutated lung cancer cells (Supplementary Figure S1A), and cPLA_2_ may be a therapeutic target for KRAS-mutated lung cancer cells. Therefore, we investigated whether MA, a proven natural inhibitor of PLA_2_, could inhibit KRAS-mutated lung cancer cells to find a new therapeutic approach for EGFR-TKI-resistant lung cancer cells.

Ca^2+^ signaling plays an important role in intracellular homeostasis and signaling cascades (Clapham, 2007; Monteith et al., 2017). Mitochondria, one of the main Ca^2+^ storage sites, play a key role in maintaining Ca^2+^ levels between the cytoplasm and the endoplasmic reticulum (ER) (Bravo-Sagua et al., 2017; Rossi et al., 2019). Studies have shown that under physiological conditions, Ca^2+^ reduces the leakage of ROS from complexes I and III of the mitochondrial respiratory chain; however, it increases the production of ROS under pathological conditions (Feissner, et al., 2009). Excess Ca^2+^ may affect mitochondrial functions, such as inhibition of oxidative phosphorylation (OXPHOS) and induction of ferroptosis (Paradies et al., 2009; Marchi et al., 2020; Nakamura et al., 2021). As an inhibitor of PLA_2_, MA is closely related to the Ca^2+^ signaling pathway and can block the entry of Ca^2+^ into cells (Wheeler et al., 1987). However, there is no detail on its function in mitochondrial Ca^2+^ storage and transport. In addition, whether the disturbance and imbalance of mitochondrial Ca^2+^ can promote the EGFR-TKI sensitivity of lung cancer cells has not yet been studied.

In this study, we investigated the effect of MA on the viability of lung cancer cells and organoids and found that MA promoted the sensitivity of KRAS-mutated lung cancer cells to osimertinib through ROS inhibition of the RAS-ERK pathway and increased the sensitivity of osimertinib-resistant lung cancer cells to osimertinib by mitochondrial Ca^2+^ overload induced ferroptosis. In conclusion, our findings suggest that the natural product MA promotes EGFR-TKI sensitivity in lung cancer cells and is a potential EGFR-TKI sensitizer.

## Materials and methods

### Cell lines, culture, and reagents

The human lung cancer cell lines A549, H157, HCC827, and PC9 were obtained from the American Type Culture Collection and were genotyped and authenticated before experiments. Cells were cultured in RPMI-1640 medium (HyClone) supplemented with 10% fetal bovine serum (ZETA) at 37°C in a humidified incubator with 5% CO2. Purified MA (>98%) (#sc-200733) was purchased from Santa Cruz. The 20 mM stock solution was made in DMSO. N-acetyl-l-cysteine (NAC) (#HY-B0215), necrostatin-1 (#HY-15760), Z-VAD-FMK (#HY-16658B), liproxstatin-1 (#HY-12726), erastin (#HY-15763) and osimertinib (#HY-15772) were purchased from MCE. The antibodies used were as follows: p-ERK (#4370), ERK (#4695), p-AMPK (#2535), AMPK (#5832), GPX4 (#52455), SLC7A11 (#12691), NRF2 (#12721), NCOA4 (#66849), FTH1 (#4393), PERK (#5683), IRE1a (#3294), LC3A/B (#12741) and GRP78 (#3177) were purchased from Cell Signaling Technology. BCL2 (#12789-1-AP), KRAS (#12063-1-AP) and GAPDH (#10494-1-AP) were purchased from Proteintech. PLA2G4A (#sc-454) was purchased from Santa Cruz.

### Cell viability, clone formation, and apoptosis assays

Cells were plated in 96-well plates at 2,000–3,000 cells per well, and after treatment with drugs for 72 h, 10 µl of CCK-8 solution was added to each well and incubated for 1.5 h. The absorbance was detected at 450 nm with a microplate reader (BioTek, Winooski, VT). For the colony formation assays, cells (800 cells per well) were seeded into 6-well plates and treated with drugs for 10 days. The medium with or without the drugs was replaced every other day. After fixation and staining with 5% crystal violet, the colonies were imaged and quantified using ImageJ software. For the apoptosis assay, cells were seeded in 6-well plates and treated with drugs for 72 h. The cells were then stained and detected by flow cytometry using an annexin V-APC apoptosis detection kit.

### ER, mitochondria, lipid droplets, ROS, lipid peroxidation staining, and detection

Cells in different treatment groups were washed once with PBS, and 200 nM ER-tracker Green FM (Beyotime), 200 nM Mito-tracker Red FM (Thermo Fisher Scientific), 1 μM BODIPY 493/503 (GLPBIO), 2.5 μM CellROX™ Deep Red (Thermo Fisher Scientific), and 2.5 μM BODIPY 581/591 C11 (Thermo Fisher Scientific) working solution were added respectively. After incubation for 30 min at 37°C, the cells were washed once with PBS. The flow cytometry or Operetta High-Content Screening System of a confocal microscope (PerkinElmer) was then used to detect ER, mitochondria, lipid droplets, ROS, and lipid peroxidation respectively.

### Iron staining and detection

Cells in different treatment groups were washed once with PBS. Cells were then stained with FerroOrange working solution (DOJINDO) at a concentration of 1 μM and incubated at 37°C for 30 min. Flow cytometry or an Operetta High-Content Screening System of a confocal microscope (PerkinElmer) was used to detect at a wavelength of Ex: 561 nm/Em: 570–620 nm.

### Ca^2+^ staining and detection

Mitochondrial Ca^2+^ was stained with Rhod-2 AM (Thermo Fisher Scientific). In brief, cells in different treatment groups were washed once with PBS and stained with 4 µM Rhod-2 AM for 30 min at 37°C. After washing with PBS 1-2 times to remove residual probes, the cells were incubated at room temperature for another 30 min to ensure complete de-esterification of AM. A flow cytometer was used to detect at a wavelength of Ex/Em = 549/578 nm. For calcium-labeled plasmid transfection, lung cancer cells were transfected with pCMV CEPIA3mt green fluorescent indicator (Addgene, #58219) and pCMV R-CEPIA1er red fluorescent indicator (Addgene, #58216) for calcium in the mitochondria and ER, respectively. The transfected cells were then treated with MA and continuously inspected by the high-content confocal microscope image analysis for 1.5 h.

### Mitochondrial respiratory oxygen consumption rate (OCR) detection

The OCR was detected according to the instructions of the Seahorse XF Cell Mito Stress Test Kit (Agilent). Briefly, on the day before the experiment, 1.5 × 10^4^ cells were seeded into each well of Seahorse XFe 24 plates and incubated in a 37°C, 5% CO_2_ incubator for 24 h. The sensor cartridge was hydrated overnight in a CO_2_-free incubator at 37°C. Seahorse XF DMEM was prepared with 10 mM glucose, 2 mM glutamine, and 1 mM sodium pyruvate. Metabolic regulation drugs were configured as 1.5 μM oligomycin, 2 µM fluorocarbonyl cyanide phenylhydrazone (FCCP), and 5 µM rotenone/antimycin A, which were respectively added to the A, B, and C dosing holes. Finally, the mitochondrial breathing program was selected for detection and analysis with the Seahorse XFe 24 Analyzer (Seahorse Bioscience).

### Western blot

Each group of cells was lysed on ice with RIPA lysis buffer for 10 min and centrifuged at 13,000 × g for 10 min to extract the total protein. A BCA assay kit was used to determine the protein concentration. The protein was added to the loading buffer and boiled at 100°C for 5 min to denature. Protein samples (30 μg) were separated by SDS‒PAGE and transferred to polyvinylidene fluoride (PVDF) membranes at 250 mA. The membrane was blocked with 5% skimmed milk at room temperature for 1 h and incubated with the primary antibody overnight at 4°C in a shaker. Goat anti-rabbit or mouse IgG was used as the secondary antibody and incubated with the membrane at room temperature for 2 h. Finally, the membranes were exposed to the ECL color solution with a chemiluminescence imager.

### LC-MS analysis of TCA intermediates

After treatment with different drugs for 48 h, cells were cultured in 1,640 medium containing 400 μM [U-^13^C16]-palmitate (Sigma) for 24 h. The cells were extracted with 80% methanol, which was prechilled at −80°C before use. In brief, the medium was removed, and the cells were washed twice with ice-cold PBS. Then, 1 mL of 80% methanol was added, and the plates were incubated for 30 min at −80°C. All the cells were quickly scraped on dry ice and re-extracted with 5 mL 80% methanol. The mixture was centrifuged at 13,000 rpm for 10 min at 4°C. The supernatant was dried in a vacuum centrifuge. Before LC-MS analysis, the metabolite residues were redissolved in 200 μL of 10% methanol.

The analysis of TCA metabolites was carried out using a Thermo Scientific Dionex Ultimate 3,000 rapid separation liquid chromatography coupled with a Q Exactive Plus high resolution-mass spectrometer. Chromatographic separation was achieved at 30°C using an HSS T3 column (2.1 × 100 mm, 2.6 µm, Waters) at a flow rate of 3 mL/min. The mobile phase was composed of A = 1% (v/v) formic acid in water and B = methanol. The ESI source was operated in negative mode. The full-scan mode was used for the acquisition of the mass spectrum with a mass resolution of 70,000 and a scan ranging from 50 to 300 m/z. The isotopic distribution of TCA intermediates was normalized to the protein concentrations of the detected cells.

### Electron microscopy imaging

The cells were fixed with 3% glutaraldehyde and 1% osmium tetroxide, and after dehydration step by step with acetone, the samples were embedded in Epon812 resin and cut into approximately 50 nm thick slices. After staining with uranyl acetate and lead citrate, images were acquired by a JEM-1400PLUS transmission electron microscope.

### RNA library construction and sequencing

Total RNA was extracted by TRIzol reagent (Invitrogen, CA, USA). Then, we performed paired-end sequencing on an Illumina sequencing platform at BIOMARKER Co., Ltd., by following the vendor’s recommended protocol.

### Organoid culture and identification

The LSL-Kras^G12D^ mouse model was obtained from the Jackson Laboratory (Sacramento, CA). Adeno-Cre (Genechem, Shanghai, China) was introduced into the trachea of mice at a dose of 1.25 × 10^11^ PFU in a total volume of 50 μL. Tumor tissues from 12-week post-infection mice were washed with cold PBS, cut into small pieces, and washed with DMEM/F12 (containing 1× Glutamine, 10 mM HEPES, and antibiotics), digested with collagenase I and IV for 0.5–1 h at 37°C. After washing twice with DMEM/F12 and centrifugation (500 g, 5 min), the dissociated cells were seeded into growth factor-reduced matrigel (Corning, #356237) at 37°C for 30 min. Next, the organoid medium, which consists of DMEM/F12 supplemented with a series of additives as described by Li et al., (Li et al., 2020a; Li et al., 2020b), was added and changed every 3 days. The lung adenocarcinoma markers TTF1, Napsin A, and the epithelial marker panCK in organoids were identified by immunohistochemistry and immunofluorescence as described by Li et al., (Li et al., 2020a; Li et al., 2020b).

### Calcein-AM staining of organoids

Organoids under drug treatment for 72 h were stained with calcein-AM (5 μM; Beyond) and Hoechst (Sigma) for 30 min at 37°C. The organoids were washed once with PBS, and images were acquired with an Operetta High-Content Screening System of a confocal microscope (PerkinElmer).

### Construction of osimertinib-resistant cell line HCC827OR

The osimertinib-resistant cell line HCC827OR was constructed by the concentration-increasing method. Specifically, cells were initially given 100 nmol/L of osimertinib, and then the concentration of osimertinib was increased until the cells were stable in 2 μmol/L of the drug-containing medium.

### Statistical analyses

The significance of differences between groups was determined using a *t*-test. Statistical analysis was performed using GraphPad Prism 8.0 Software. *p*-value< 0.05 was considered statistically significant.

## Results

### MA inhibits the proliferation of lung cancer cells and organoids

**FIGURE 1:**
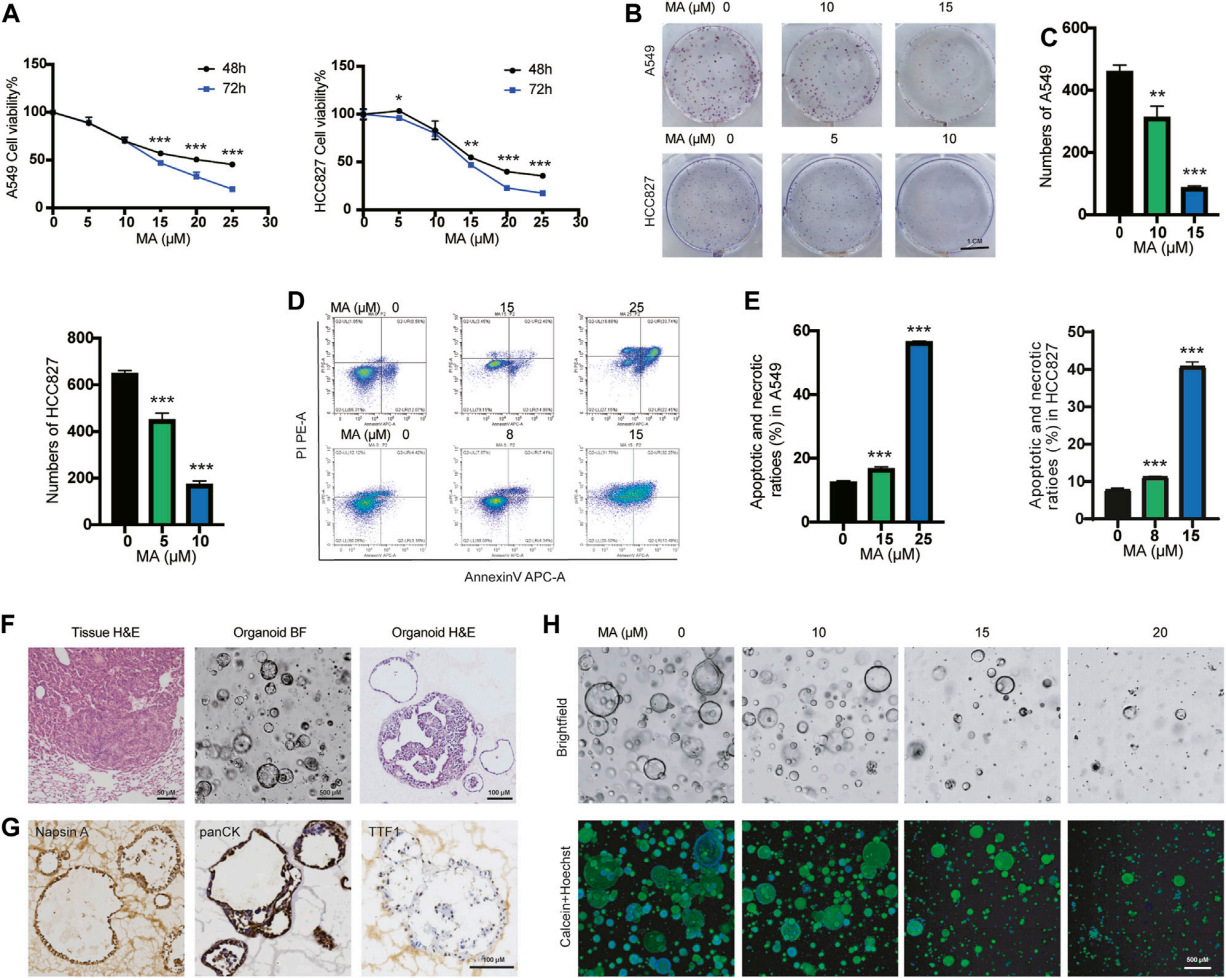
MA inhibits the proliferation of lung cancer cells and organoids. **(A)** The viability of A549 and HCC827 cells were examined by CCK8 assay after treatment with different concentrations of MA for 48 and 72 h. **(B)** Representative results of crystal violet staining for colony formation. **(C)** Quantitative analysis of the colony number of A549 and HCC827 cells. **(D)** Representative results of annexin V/PI staining. **(E)** Quantitative analysis of apoptosis in A549 and HCC827 cells. **(F)** H&E staining of tumor tissue from the KRAS^G12D^ mouse model, as well as representative bright field (BF) microscopy images and H&E staining of organoids. **(G)** Immunohistochemistry analysis of lung adenocarcinoma markers TTF1, Napsin A, and the epithelial marker panCK in organoids. **(H)** Bright field and Calcein staining of organoids after treatment with different concentrations of MA. *p < .05, **p < .01, ***p < .001.

To investigate the inhibitory effects of MA in lung cancer cells, we treated different types of lung cancer cells, including KRAS-mutated lung cancer cell lines A549 and H157 and EGFR-mutated lung cancer cell lines HCC827 and PC9, with different concentrations of MA for 48 and 72 h. Cell counting kit-8 (CCK-8) assays showed that MA inhibited the proliferation of four types of lung cancer cells in a concentration- and time-dependent manner (Figure 1A; Supplementary Figure S1B). At the same time, colony formation experiments showed that MA suppressed the colony formation efficiency of lung cancer cells (Figures 1B, C; Supplementary Figures S1C, D). Wang et al., found that MA promoted the apoptosis of oral cancer cells (Wang et al., 2019), so we also detected whether MA could induce apoptosis in lung cancer cells. As a result, MA treatment potently promoted the apoptosis of several lung cancer cell lines in a dose-dependent manner (Figures 1D, E; Supplementary Figures S1E, F) and downregulated the antiapoptotic protein of BCL2 (Supplementary Figure S1G). To establish the *in vitro* drug screening system, we constructed primary KRAS-mutated lung cancer organoids from a mouse model of Kras^G12D^-driven lung cancer (Figure 1F). To further characterize the organoids, we performed immunohistochemistry and immunofluorescence analysis of two adenocarcinoma markers, thyroid transcription factor (TTF-1), Napsin A, as well as the epithelial marker panCK (Li et al., 2020a). The organoids showed positive staining of TTF-1, Napsin A, and panCK (Figure 1G; Supplementary Figure S1H), suggesting the consistency with original tumor tissues. After treating organoids with MA for 72 h, calcein (viable cell dye) staining showed that MA significantly inhibited organoid spheroidization in a dose-dependent manner (Figure 1H), indicating that MA inhibited the stemness of KRAS-mutated lung cancer cells. Taken together, these results showed that MA significantly reduced the viability and proliferation of KRAS-mutated lung cancer cells and organoids, indicating that MA is a potential suppressor of KRAS-mutated lung cancer.

### MA causes ER vacuolation through oxidative stress

**FIGURE 2:**
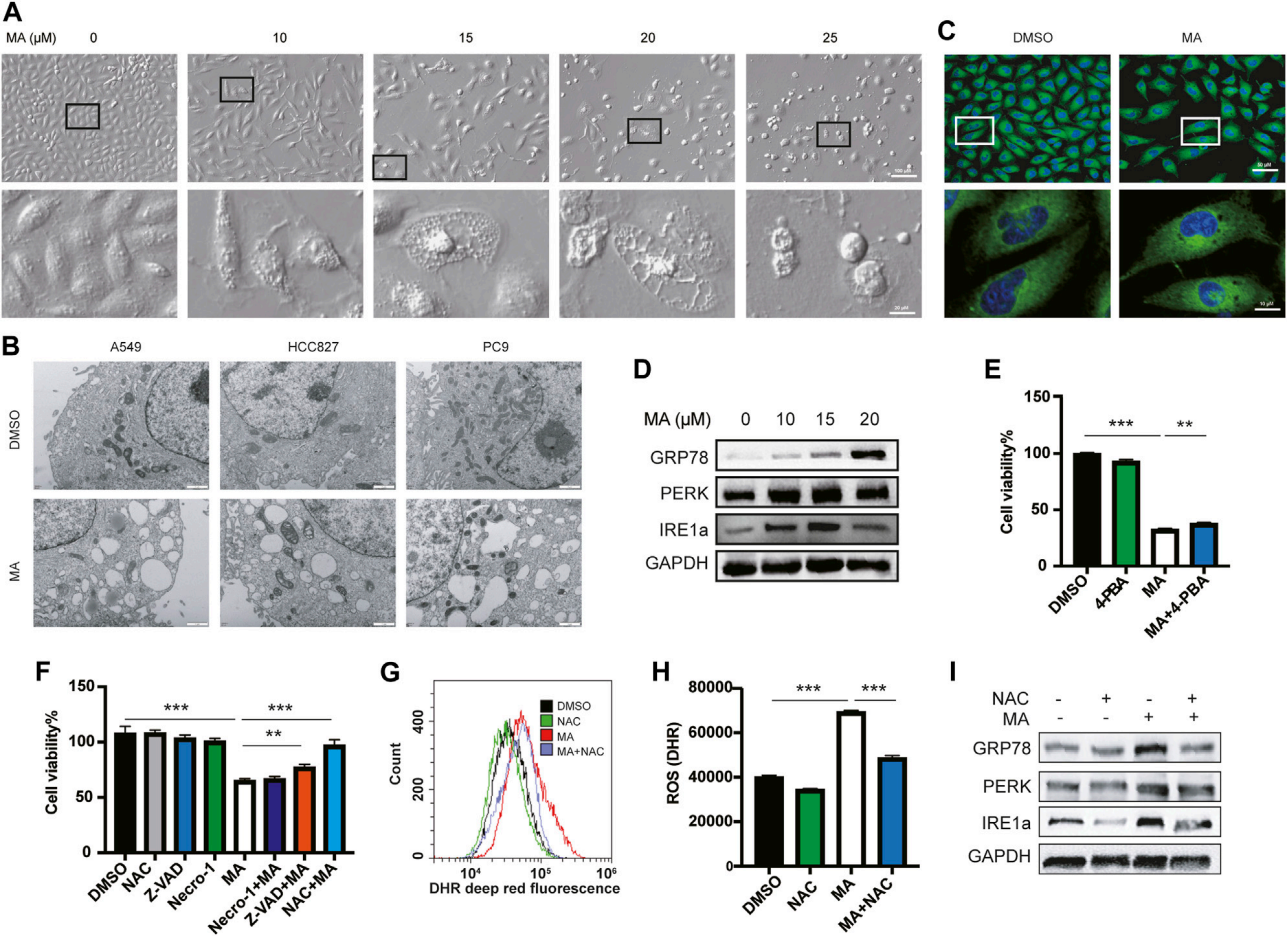
MA causes ER vacuolation through oxidative stress. **(A)** Representative morphological changes in A549 cells treated with different concentrations of MA. **(B)** Electron microscope analysis of A549, HCC827, and PC9 cells treated with MA. **(C)** ER-tracker staining of A549 cells. **(D)** MA-activated ER stress-related markers were examined by western blot. **(E)** 4-PBA alleviated MA-suppressed cell proliferation, as shown by CCK-8 detection. The concentration of 4-PBA and MA were 500 μM and 15 μM, respectively. **(F)** Cell viability analysis of A549 cells co-treated with MA and several cell death inhibitors. Representative flow cytometry histogram **(G)** and quantification of DHR (ROS marker, deep red) **(H)** in A549 cells treated with single MA (15 μM) or a combination of MA and NAC (1 mM). **(I)** As shown by western blot analysis, NAC inhibited MA-increased expression of ER stress-related markers. **p < .01, ***p < .001.

After treating A549 cells with different concentrations of MA, we found that the morphology of A549 cells changed significantly with the formation of multiple cytoplasmic vacuoles, and the number of vacuoles increased with higher concentrations of MA treatment (Figure 2A). Drug-induced cytoplasmic vacuoles in cancer cells is a form of cell death and are associated with ER (Schoeman et al., 2020), lysosomes (Hino et al., 2020), as well as macropinocytosis derived from endosomes (Overmeyer et al., 2011). To determine the mechanism of cell vacuolization induced by MA, we further observed a more detailed organelle morphology with electron microscopy and found that a large number of vacuolar structures originating from the ER appeared in lung cancer cells after treatment with MA (Figure 2B). Similar vacuolar morphology was also observed after staining with ER-tracker (Figure 2C). Therefore, we speculated that MA induced a large amount of abnormal vacuolization in the ER and caused ER stress in lung cancer cells. We further examined the expression of several ER stress markers, and the results showed that MA increased the expression of GRP78, PERK, and IRE1a in a concentration-dependent manner (Figure 2D). Furthermore, we co-treated A549 cells with the ER stress inhibitor 4-PBA and MA and found that 4-PBA alleviated MA-suppressed cell proliferation; however, its recovery was limited (Figure 2E). To further determine the main role of MA-induced cell death, we co-treated lung cancer cells with MA (15 μM) and several cell death inhibitors. The results indicated that treatment with Z-VAD-FMK (a pan-caspase inhibitor, 10 μM) alleviated MA-induced cell death to a certain extent and that nercostatin-1 (a potent inhibitor of necroptosis, 10 μM) did not protect against MA-induced cell death, while N-acetyl-l-cysteine (NAC) (the ROS inhibitor, 1 mM) almost completely rescued MA-induced lung cancer cell death (Figure 2F; Supplementary Figure S2A). Therefore, it can be seen that the main role of MA in lung cancer cells may be caused by oxidative stress. We further detected the ROS levels after treatment with MA in lung cancer cells. As expected, ROS accumulation was significantly increased following treatment with MA, while co-treatment with NAC decreased MA-induced ROS levels (Figures 2G, H). To determine whether MA-induced ER stress was also caused by ROS, we examined the expression of ER stress markers after co-treatment with NAC and MA. The results showed that NAC downregulated the expression of GRP78, PERK, and IRE1, which was increased by MA (Figure 2I). Taken together, the above results suggest that MA inhibited proliferation and triggered ER stress in lung cancer cells mainly through ROS.

### MA induces mitochondrial ROS elevation and lipid peroxidation

**FIGURE 3:**
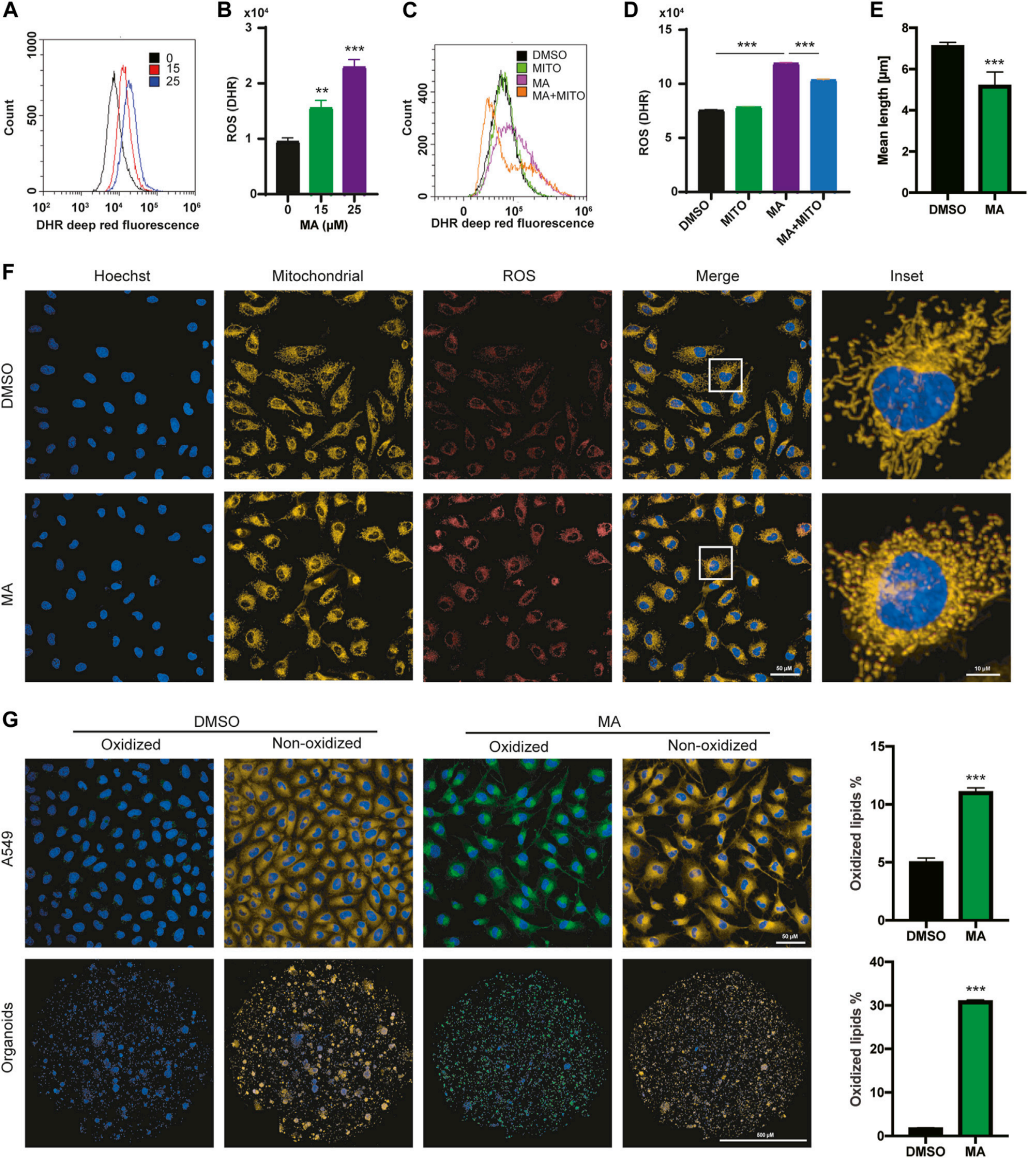
MA induces mitochondrial ROS elevation and lipid peroxidation. Representative flow cytometry histogram **(A)** and quantification of DHR (ROS marker, deep red) **(B)** in A549 cells treated with different concentrations of MA. Representative flow cytometry histogram **(C)** and quantification of DHR (ROS marker, deep red) **(D)** in A549 cells treated with mitoTEMPO (1 μM) and MA (15 μM). **(E)** Quantification of mitochondrial length in A549 cells treated with MA (15 μM). **(F)** Representative confocal fluorescence microscopy images of mitochondria and ROS. **(G)** Representative confocal fluorescence microscopy images and quantifications of lipid peroxidation staining with BODIPY 581/591 C11 in A549 cells and organoids treated with MA (15 μM). **p < .01, ***p < .001.

To further study the relationship between MA and the increase in ROS levels in lung cancer cells, we detected the ROS levels after treatment with different concentrations of MA. The results showed that ROS levels increased significantly in a dose-dependent manner in A549 (Figures 3A, B), H157 (Supplementary Figures S3A, B), and HCC827 (Supplementary Figures S3C, D) cells. Since the main source of cellular ROS is mitochondria (Yang et al., 2016; Moloney and Cotter, 2018), to clarify whether the ROS induced by MA are also derived from mitochondria, we co-treated A549 (Figures 3C, D) and HCC827 (Supplementary Figures S3E, F) cells with MA and mitoTEMPO (a mitochondrial-targeted antioxidant). The results indicated that mitoTEMPO significantly suppressed MA-induced ROS levels. Moreover, we costained A549 cells and HCC827 cells with MitoTracker and CellROX (a ROS indicator) and found that MA-induced ROS and mitochondria were almost completely coincident (Figure 3F; Supplementary Figure S3G), indicating a mitochondrial source of ROS. Notably, MA treatment impaired the mitochondrial morphology of lung cancer cells, reducing mitochondrial length (Figure 3E; Supplementary Figure S3H). Studies have shown that increasing ROS levels are a cause of lipid peroxidation (Su et al., 2019). To investigate whether MA induces lipid peroxidation, we stained MA-treated A549 (Figure 3G), H157, and HCC827 cells (Supplementary Figure S3I) with BODIPY 581/591 C11 (a lipid peroxidation sensor). The results revealed that MA significantly promoted lipid peroxidation in lung cancer cells. Moreover, confocal microscopy analysis in KRAS-mutated primary lung cancer organoids also showed that MA increased lipid peroxidation in organoids (Figure 3G). Overall, these results indicated that MA increased mitochondrial ROS and lipid peroxidation in lung cancer cells.

### MA reduces mitochondrial oxidative metabolism and promotes the accumulation of lipid droplets

**FIGURE 4:**
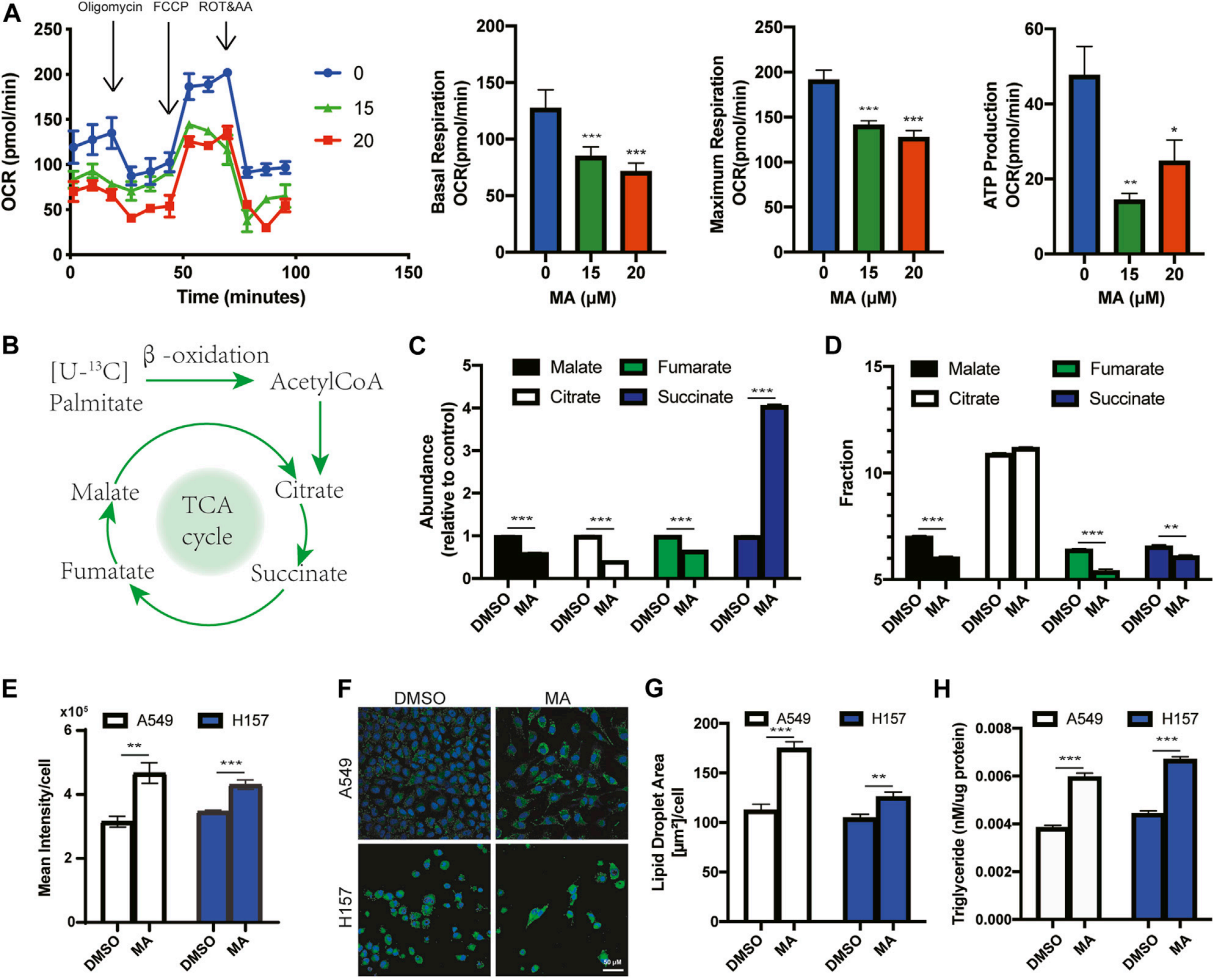
MA reduces mitochondrial oxidative metabolism and promotes the accumulation of lipid droplets. **(A)** Oxygen consumption rate (OCR) in A549 cells treated with MA. **(B)** Schematic diagram of palmitic acid oxidation into intermediates of the TCA cycle. **(C)** The abundance of TCA intermediates in A549 cells treated with MA (15 μM). **(D)** ^13^C fraction of TCA intermediates in A549 cells treated with MA (15 μM). **(E)** Lipid droplet quantification by flow cytometry of A549 and H157 cells treated with MA (15 μM). **(F)** Representative confocal fluorescence microscopy images of lipid droplet staining with BODIPY 493/503 (green) in A549 cells and H157 cells treated with MA (15 μM). **(G)** Quantification of the lipid droplet area per cell in A549 and H157 cells. **(H)** Triacylglycerols were measured in A549 and H157 cells treated with MA (15 μM). *p < .05, **p < .01, ***p < .001.

ROS accumulation is accompanied by impaired mitochondrial function (Yang et al., 2016; Moloney and Cotter, 2018). To assess mitochondrial function, we detected the mitochondrial oxygen consumption rate (OCR), a marker of mitochondrial respiratory capacity and energy production, after MA treatment in lung cancer cells. We found that basal respiration, maximal respiration, and mitochondrial ATP production were significantly reduced in MA-treated lung cancer cells, suggesting strong mitochondrial dysfunction triggered by MA (Figure 4A). Mitochondria are the main sites for fatty acid oxidation (FAO) (Rossi et al., 2019). To determine whether MA-reduced mitochondrial oxidative metabolism could decrease the mitochondrial capacity to oxidize lipids, we detected FAO by mass spectrometry to trace the fraction of ^13^C-labeled palmitic acid into intermediates of the TCA cycle (Figure 4B). The results showed that citric acid, malate, and fumarate were significantly inhibited by MA, while succinate potently increased by approximately 4-fold in A549 cells and also increased in H157 cells upon MA treatment (Figure 4C; Supplementary Figure S4A). Since succinate accumulation is associated with ROS production (Kamarauskaite et al., 2020), the accumulation of succinate might be one of the reasons for MA-induced ROS elevation. Further analysis of the ^13^C-labeled intermediates showed that the proportion of ^13^C in malate, fumarate, and succinate was significantly reduced in MA-treated lung cancer cells (Figure 4D; Supplementary Figure S4B), indicating that MA suppressed the metabolic flux of ^13^C-labeled palmitic acid into the TCA cycle, that is, inhibited FAO. Since lipid droplets (LDs) are sources of mitochondrial FAO (Bosch et al., 2020), we further examined the effect of MA on LDs. Staining of LDs with BODIPY 493/503 and measurement by flow cytometry showed that MA significantly promoted the accumulation of LDs in several lung cancer cells (Figure 4E; Supplementary Figure S4C). Confocal microscopy showed that this was due to an increase in LD size upon MA treatment (Figures 4F, G; Supplementary Figures S4D, E). Moreover, we quantified the total amount of triacylglycerols (TAGs), the main component of LDs, and found that MA treatment resulted in a significant increase in TAGs (Figure 4H), which further proved the promoting effect of MA on LDs. Electron microscopy also showed more LDs in MA-treated lung cancer cells than in control cells (Supplementary Figure S4F). These results suggest that MA impairs mitochondrial oxidative metabolism and fatty acid catabolism, leading to the accumulation of TAGs in LDs.

### MA triggers ferroptosis by inducing mitochondrial Ca^2+^ overload in lung cancer cells

**FIGURE 5:**
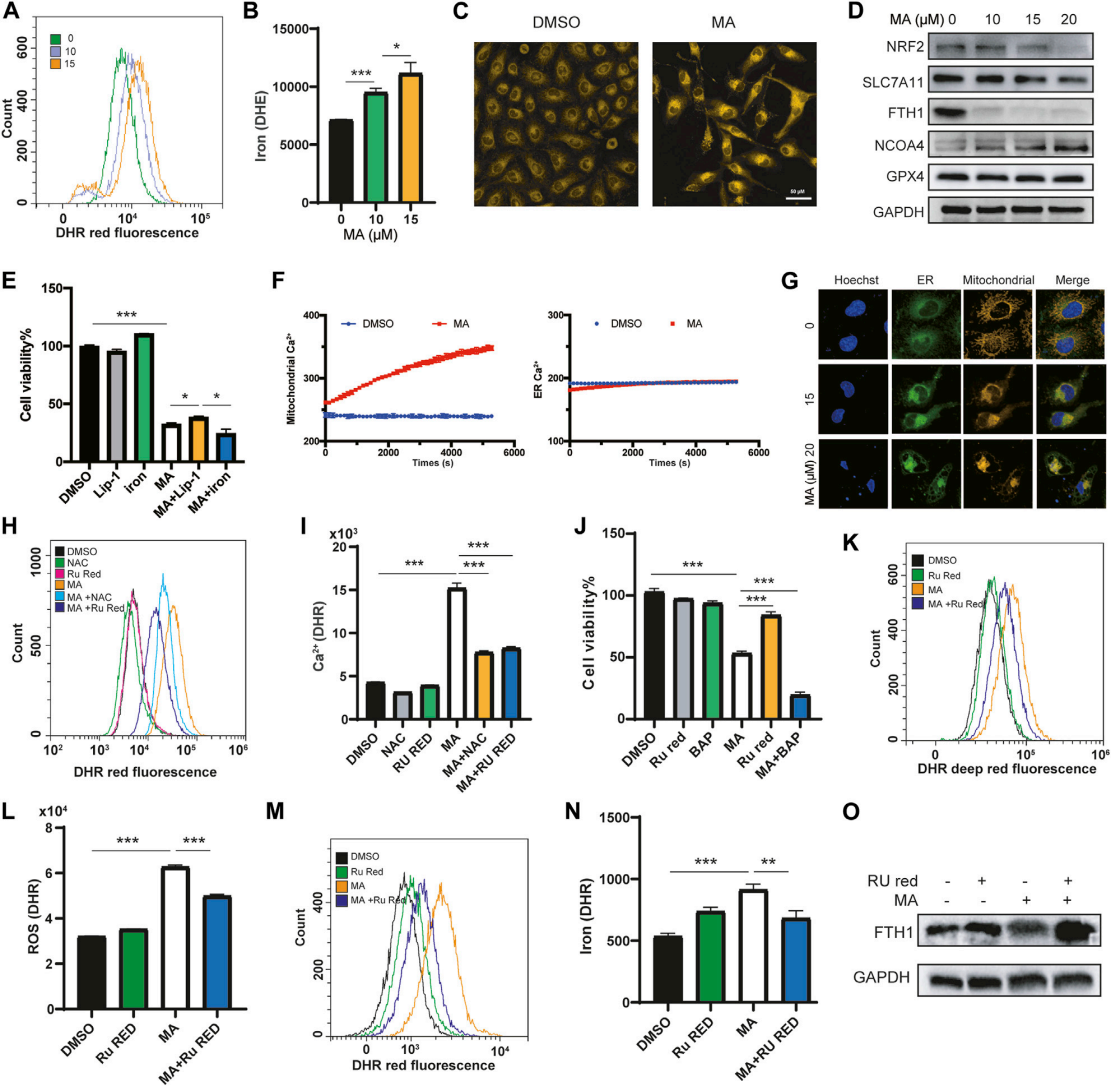
MA triggers ferroptosis by inducing mitochondrial Ca^2+^ overload in lung cancer cells. Representative flow cytometry histogram **(A)** and quantification of iron fluorescence intensity with FerroOrange staining **(B)** in A549 cells treated with different concentrations of MA. **(C)** Representative confocal fluorescence microscopy images of iron staining with ferroOrange (red) in A549 cells treated with MA (15 μM). **(D)** Ferroptosis markers were analyzed by western blot in A549 cells treated with different concentrations of MA. **(E)** Cell viability of A549 cells cotreated with MA (15 μM) and Lip-1 (1 μM) or ammonium iron (III) citrate (.1 mg/ml) by CCK-8 assays. **(F)** Mitochondrial and ER Ca^2+^ response in cells treated with MA (15 μM) for 1.5 h. **(G)** Representative confocal fluorescence microscopy images of mitochondrial and ER staining with MitoTracker (red) and ER-Tracker (green) in A549 cells treated with different concentrations of MA. Representative flow cytometry histogram **(H)** and quantification of mitochondrial Ca^2+^ fluorescence intensity with RhoA staining **(I)** in A549 cells treated with MA (15 μM), NAC (1 mM), and Ru Red (1.5 μM). **(J)** Cell viability of A549 cells treated with MA (15 μM), BAP (1 μM), and Ru Red (1.5 μM). Representative flow cytometry histogram **(K)** and quantification of ROS fluorescence intensity **(L)** with CellROX staining in A549 cells treated with MA (15 μM) and Ru Red (1.5 μM). Representative flow cytometry histogram **(M)** and quantification of iron fluorescence intensity **(N)** with FerroOrange staining in A549 cells treated with MA (15 μM) and Ru Red (1.5 μM). **(O)** Ru Red restored the MA-inhibited abundance of FTH1. *p < .05, **p < .01, ***p < .001.

ROS accumulation, lipid peroxidation, enlarged mitochondrial cristae, and iron overload are critical evidence of ferroptosis (Stockwell, 2022; Stockwell, 2022). Electron microscopy revealed that MA treatment resulted in mitochondrial matrix condensation and enlarged cristae (Figure 2B; Supplementary Figure S4F). We further detected the iron concentration in lung cancer cells after treatment with MA by using the Fe^2+^ fluorescent indicator FerroOrange. As expected, MA treatment significantly increased the iron content in a dose-dependent manner detected by flow cytometry (Figures 5A, B). Confocal microscopy detection also showed that MA promoted iron accumulation in lung cancer cells (Figure 5C). Moreover, the expression of positive regulatory proteins for ferroptosis, including nuclear factor erythroid 2-related factor 2 (NRF2), solute carrier family 7 member 11 (SLC7A11) and ferritin heavy chain (FTH1), significantly decreased after treatment with MA, while the expression of nuclear receptor coactivator 4 (NCOA4) increased. Nevertheless, MA treatment did not affect the expression of glutathione peroxidase 4 (GPX4) (Figure 5D). In addition, the ferroptosis inhibitor liproxstatin-1 (Lip-1) moderately blocked MA-induced cell death, while additional iron from ammonium iron (III) citrate further inhibited cell viability (Figure 5E). These findings strongly indicated that MA induced ferroptosis in lung cancer cells. Since NCOA4 is responsible for the delivery of ferritin to the lysosome *via* autophagosomes (Hassannia et al., 2019), we further investigated the expression of the autophagy marker LC3I/II and found that MA induced LC3II expression in a dose-dependent manner (Supplementary Figure S5A). NRF2 is a master regulator of antioxidant transcription factors that protect against lipid peroxidation and ferroptosis by increasing the transcription of multiple cytoprotective enzymes, such as SLC7A11 (DeNicola et al., 2011). These mechanisms revealed that MA might induce ferroptosis through the NRF2-SLC7A11 and NCOA4-FTH1 pathways.

Studies have shown that mitochondrial Ca^2+^ overload is closely related to ferroptosis (Chen et al., 2020; Nakamura et al., 2021), and MA, as a PLA_2_ inhibitor, is related to calcium signaling (Wheeler et al., 1987). Therefore, we speculated that MA affected the mitochondrial Ca^2+^ concentration to induce ferroptosis. We transfected lung cancer cells with mitochondrial or ER calcium-labeled plasmids and analyzed the calcium levels in mitochondria and ER after treatment with MA for 1.5 h by high-content confocal microscopy. The results indicated that the Ca^2+^ level in the ER did not change in a short time, while the mitochondrial Ca^2+^ concentration increased approximately 1.4-fold after treatment with MA for 1.5 h (Figure 5F). The main source of mitochondrial Ca^2+^ comes from the cytosol and ER (Bravo-Sagua et al., 2017; Rossi et al., 2019). To further clarify the crosslink between the ER and mitochondria, costaining with mito-tracker and ER-tracker fluorescence probes showed that the cross-linked area increased with MA treatment in lung cancer cells (Figure 5G). To investigate whether Ca^2+^ signaling was a key determinant of MA-induced ferroptosis, we further treated lung cancer cells with ruthenium red (Ru Red, an inhibitor of mitochondrial Ca^2+^ uptake), and the mitochondrial Ca^2+^ concentration was assessed by detecting the fluorescence intensity of the mitochondrial Ca^2+^ indicator Rhod-2 with a flow cytometer. The results showed that MA treatment significantly increased the mitochondrial Ca^2+^ concentration; however, Ru Red and NAC restored MA-induced mitochondrial Ca^2+^ overload (Figures 5H, I; Supplementary Figures S5B, C), indicating that MA induced mitochondrial Ca^2+^ overload by ROS accumulation. Interestingly, cotreatment with Ru Red and MA recovered cell viability, while BAPTA (BAP, a cytoplasmic calcium chelator) further exacerbated MA-induced cell death (Figure 5J), suggesting that MA induced mitochondrial calcium overload but decreased cytoplasmic calcium. Moreover, Ru Red reduced MA-induced ROS levels (Figures 5K, L; Supplementary Figures S5D, E), revealing the interaction between ROS and mitochondrial Ca^2+^. Notably, Ru Red also suppressed the MA-induced accumulation of iron (Figures 5M, N; Supplementary Figures S5F, G). Further detection of ferroptosis pathway proteins by western blotting revealed that Ru Red significantly restored the expression of FTH1 (Figure 5O), which plays a major role in iron sequestration, detoxification, and storage (Muhoberac and Vidal, 2019). These results suggested that mitochondrial Ca^2+^ overload promoted FTH1-mediated iron concentration. All the above results indicated that MA induced ferroptosis in lung cancer cells, which was caused by NRF2-SLC7A11 and mitochondrial Ca^2+^ overload-induced FTH1 pathways.

### The combination of MA and osimertinib inhibits EGFR-TKI resistance in lung cancer cells

**FIGURE 6:**
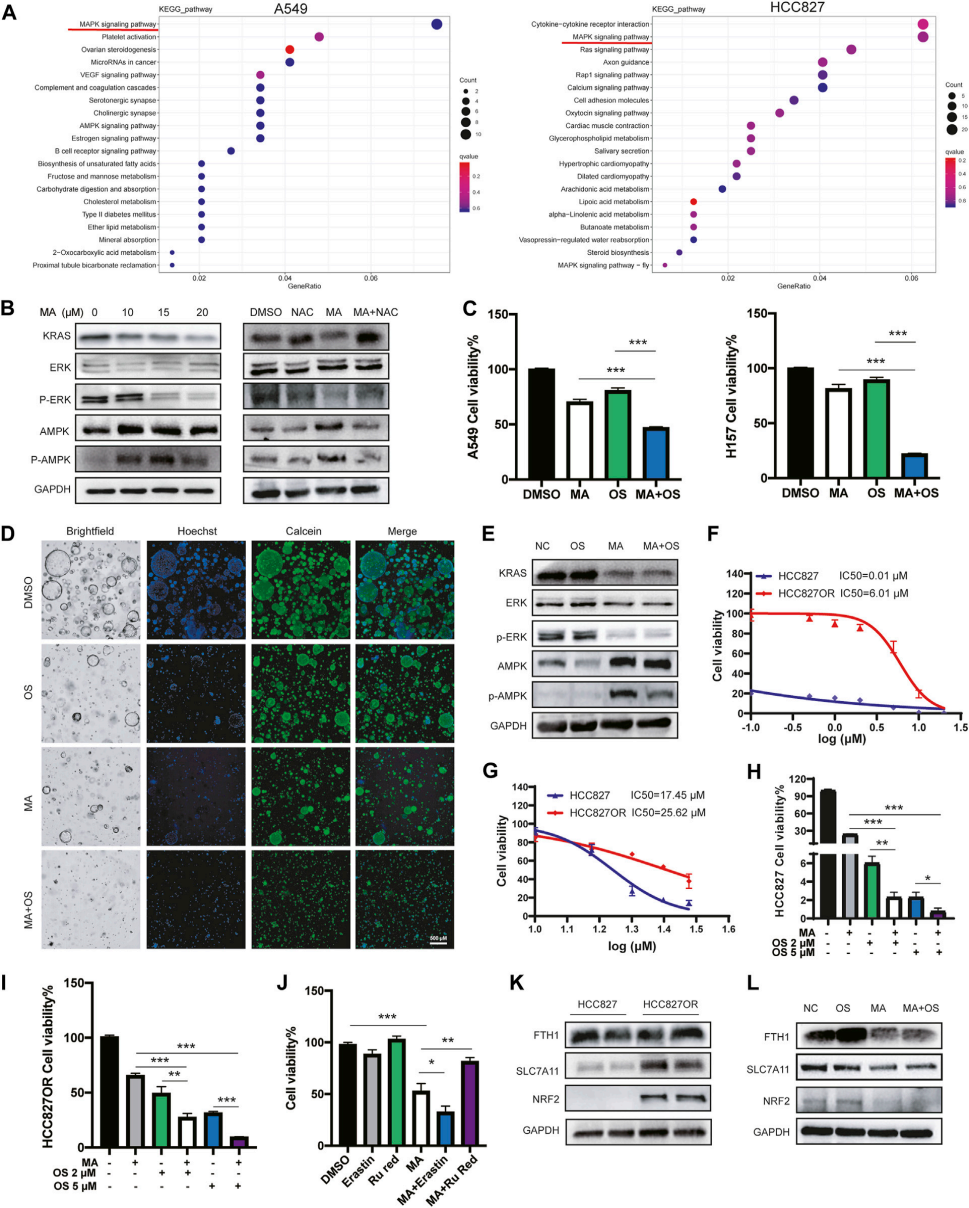
The combination of MA and osimertinib inhibits EGFR-TKI resistance in lung cancer cells. **(A)** Heatmap of KEGG pathway enrichment analyses in A549 and HCC827 cells. **(B)** The expression of p-ERK/ERK, p-AMPK/AMPK, and KRAS in A549 cells treated with MA and NAC (1 mM). **(C)** Cell viability of A549 and H157 cells treated with MA (10 μM) and osimertinib (1 μM). **(D)** Cell viability of organoids treated with MA (15 μM) and osimertinib (2 μM). **(E)** The expression of p-ERK/ERK, p-AMPK/AMPK, and KRAS in A549 cells treated with MA (15 μM) and osimertinib (2 μM). Viability of HCC827 and HCC827OR cells treated with different concentrations of osimertinib **(F)** or MA **(G)**. Viability of HCC827 **(H)** and HCC827OR **(I)** cells treated with MA (10 μM) and osimertinib. **(J)** Cell viability of HCC827OR cells treated with MA (15 μM), erastin (1 μM), and Ru Red (1.5 μM). **(K)** The abundance of FTH1, SLC7A11, and NRF2 in HCC827 and HCC827OR cells. **(L)** The expression of FTH1, SLC7A11, and NRF2 in HCC827OR cells treated with MA (15 μM) and osimertinib (2 μM). *p < .05, **p < .01, ***p < .001.

To further explore the underlying molecular mechanism by which MA inhibits the proliferation of lung cancer cells, we performed RNA sequencing to profile the transcriptomes of A549 and HCC827 cells treated with MA. KEGG pathway enrichment analyses revealed that the MAPK signaling pathway was the top-ranked pathway affected by MA in lung cancer cells (Figure 6A). It is well known that the MAPK pathway includes a small G protein (RAS) and three protein kinases (RAF, MEK, and ERK) (Guo et al., 2020). Therefore, the expressions of KRAS and p-ERK/ERK were detected by western blotting and the results exhibited a dose-dependent decrease with MA treatment, indicating the suppression of the KRAS-ERK signaling pathway by MA treatment. To examine whether MA affects the MAPK pathway through ROS triggered by MA, we cotreated cells with the ROS inhibitor NAC and MA. As a result, NAC increased the MA-suppressed expression of KRAS and p-ERK (Figure 6B), indicating that the inhibition of the KRAS-ERK pathway by MA was ROS-dependent. Since cells harboring KRAS mutation are the main reason for EGFR-TKI resistance with the continuous expression of KRAS, we speculated that the combination treatment of MA and EGFR-TKI osimertinib was a strategy to inhibit KRAS-mutated lung cancer cells. The results showed that the combination treatment of MA and osimertinib decreased the proliferation of KRAS-mutated lung cancer cells of A549 and H157 compared to the osimertinib treatment group at a low dose (1 μM) (Figure 6C; Supplementary Figure S6A). In addition, the combination of MA and osimertinib also inhibited KRAS-mutated lung cancer organoids compared to single-drug treatment groups (Figure 6D). It was reported that the activation of the AMPK pathway may overcome the drug resistance induced by KRAS mutation in CRC (Ye et al., 2020) and that ROS activated the KRAS/AMPK pathway (Zhao et al., 2019). Therefore, we further detected the expression of p-AMPK/AMPK after treatment with MA. The results indicated that MA triggered AMPK expression and that it was also ROS dependent (Figure 6B). Western blotting further showed that KRAS and p-ERK decreased, while AMPK increased in the MA and osimertinib combination group compared to the osimertinib treatment alone group in A549 cells (Figure 6E).

In addition, an *in vitro* cell model of acquired resistance to osimertinib was established in EGFR mutant lung cancer HCC827 cells (HCC827OR), the IC50 values of which were over 500-fold higher than those of the parental cells (Figure 6F). To investigate whether MA also increased the sensitivity of HCC827OR cells to osimertinib, a CCK-8 assay showed that HCC827OR cells were also more resistant to MA than the parental HCC827 cells (Figure 6G), but the combination of MA and osimertinib significantly inhibited the viability of HCC827 (Figure 6H) and HCC827OR (Figure 6I) cells, suggesting that MA overcame acquired resistance to EGFR-TKIs in lung cancer cells. Studies have shown that drug-resistant cells are closely related to ferroptosis (Huang et al., 2021; Ma et al., 2021; Zhang et al., 2021). We further treated HCC827OR cells with the ferroptosis inducer erastin and found that MA promoted the ferroptosis sensitivity of drug-resistant cells, while Ru Red restored MA-triggered cell death (Figure 6J). Moreover, western blot analysis revealed upregulation of NRF2 and SLC7A11 in HCC827OR cells compared to the parental sensitive HCC827 cells (Figure 6K), indicating that osimertinib resistance is related to the ferroptosis pathway. Combination treatment with MA and osimertinib further inhibited the expression of NRF2 and SLC7A11 compared to single drug-treated groups (Figure 6L). Although there was no obvious difference in the expression of FTH1 in HCC827 and HCC827OR cells, the expression of FTH1 was significantly inhibited in the MA and OS cotreatment group compared with the OS treatment alone group (Figure 6L) and the same results were found in A549 cells (Supplementary Figure S6B). Previous reports have indicated that KRAS mutation significantly promotes the expression of NRF2 and then activates SLC7A11 (Hu et al., 2020). Knockdown of KRAS suppresses NRF2 activity (Gwinn et al., 2018). Therefore, MA might depress the NRF2-SLC7A11 signaling by inhibiting the expression of KRAS. The above results indicate that MA alleviates the resistance of lung cancer cells to osimertinib, mainly by inhibiting the KRAS-ERK pathway, as well as the KRAS-NRF2-SLC7A11 and FTH1 ferroptosis axis.

## Discussion

Strategies for lung cancer patients with KRAS mutations and EGFR-TKI resistance are limited, and it is imperative to develop new EGFR-TKI sensitizers and combination strategies to overcome resistance to EGFR-TKIs. In this study, we investigated the effect of MA on EGFR-TKI-resistant lung cancer cells. The results showed that MA inhibited the proliferation of lung cancer cells and organoids. Moreover, MA induced ER stress and mitochondrial dysfunction *via* ROS oxidative stress. Combination treatment with MA and osimertinib improved the sensitivity of lung cancer cells to EGFR-TKIs through ROS suppression of the KRAS-ERK signaling pathway, as well as NRF2-SLC7A11 axis inhibition- and mitochondrial Ca^2+^ overload-triggered ferroptosis. Overall, MA is a potential EGFR-TKI sensitizer.

KRAS mutation is an important driver gene of NSCLC. The incidence of KRAS mutation in Western populations reaches 20%–25%, and in Asian populations, it also reaches 10%–15% (Reck et al., 2021). It is usually associated with poor prognosis and drug resistance. Continued activation of KRAS and the downstream of mitogen-activated protein kinase (MAPK) signaling is a common mechanism of resistance to osimertinib and other EGFR-TKIs (Samatar and Poulikakos, 2014; Zhang et al., 2021). Strategies to inhibit KRAS have been hindered due to the lack of a proper binding pocket for small molecules. Moreover, inhibiting the downstream effectors of KRAS showed modest or no clinical responses due to the compensatory activation of alternative pathway effectors (Ambrogio et al., 2018). Therefore, it has become a new hotspot to seek more upstream regulatory strategies for KRAS to overcome the resistance of KRAS-mutated patients to EGFR-TKIs. Phospholipase A2s (PLA2s) are key enzymes that catalyze the hydrolysis of membrane phospholipids to release bioactive lipids such as arachidonic acid which play an important role in inflammation and cancer (Peng et al., 2021). Studies have shown that targeting cPLA2 inhibits gastric cancer and augments chemotherapy efficacy by suppressing the Ras/MEK/ERK and Akt/β-catenin pathways (Liao et al., 2021), which indicates that PLA2 may be one of the upstream regulations of RAS pathways. Our study also found that MA, acting as a PLA2 inhibitor, significantly inhibited KRAS expression and the downstream ERK pathway in lung cancer cells, suggesting that MA may inhibit KRAS by suppressing PLA2 and overcome the EGFR-TKI resistance in KRAS- mutated lung cancer cells.

Ferroptosis is an emerging type of cell death induced by metallic iron and ROS-induced lipid peroxidation. It has been reported that ferroptosis can be used to overcome resistance to targeted therapy. After acquiring resistance to EGFR-TKIs, EGFR-mutated lung cancer cells showed increased sensitivity to ferroptosis-inducing agents (Ma et al., 2021). NRF2 was upregulated in EGFR-TKI-resistant cells, and NRF2 activation induced resistance to EGFR-TKIs, which was reversed by the inhibition of GPX4 and SOD2 (Ma et al., 2021). SLC7A11, the downregulation of NRF2, is introduced into cystine for the synthesis of the antioxidant peptide glutathione (GSH) (Koppula et al., 2018), which inhibits lipid peroxidation and ferroptosis (Huang et al., 2021). The histone deacetylase inhibitor vorinostat promotes ferroptosis in EGFR-mutant lung adenocarcinoma cells by inhibiting SLC7A11 (xCT) and enhancing the efficacy of ferroptosis inducers (Zhang et al., 2021). Therefore, NRF2 and SLC7A11 may be potential therapeutic targets for overcoming resistance to EGFR-TKIs. In our study, by constructing osimertinib-resistant HCC827OR cells, it was found that the expression of NRF2 and SLC7A11 was significantly increased. After MA treatment, the NRF2-SLC7A11 ferroptosis signaling axis was inhibited and increased the sensitivity to osimertinib, suggesting that MA-induced ferroptosis through the NRF2-SLC7A11 axis to overcome resistance to EGFR-TKIs.

With the deepening of ferroptosis research, it was found that mitochondrial calcium overload and ferroptosis are closely related. Peng Chen et al. found that the natural product erianin exerts its anti-inflammatory properties by inducing calcium/calmodulin-dependent ferroptosis and inhibiting the metastasis of lung cancer cells (Chen et al., 2020). The mitochondrial Ca^2+^ uptake regulator mitochondrial calcium uptake 1 (MICU1) is involved in cold stress-induced ferroptosis. Activation of mitochondrial Ca^2+^ signaling promotes cystine addiction and sensitizes PDAC cells to ferroptosis (Nakamura et al., 2021). However, there was no report on the specific mechanism of mitochondrial Ca^2+^ imbalance-induced ferroptosis. Our study found that MA induced mitochondrial Ca^2+^ overload while inhibiting the expression of the ferritin gene FTH1. Interestingly, when MA-induced mitochondrial Ca^2+^ was inhibited with Ru Red, the expression of FTH1 and the iron content also decreased, suggesting that MA might inhibit FTH1-mediated iron concentration by promoting mitochondrial Ca^2+^ overload. In addition, previous studies on ferroptosis and EGFR-TKI resistance have focused on NRF2 and SLC7A11, there have been no studies on whether FTH1 can be used as a drug resistance target. We found that co-treatment with MA and osimertinib in lung cancer cells significantly inhibited the expression of FTH1 compared with the osimertinib alone treatment group, which further activated the ferroptosis pathway and enhanced EGFR-TKI sensitivity.

In conclusion, our study shows that MA is a novel EGFR-TKI sensitizer in KRAS-mutated and osimertinib-resistant lung cancer cells by suppressing the KRAS-ERK pathway and inducing ferroptosis *via* suppressing NRF2-SLC7A11 axis and mitochondrial Ca^2+^ overload induced-FTH1 pathways.

## Data Availability

The datasets presented in this study can be found in online repositories. The names of the repository/repositories and accession number (s) can be found in the article/Supplementary Material.
