# Supplementary figures and images for: Natural product manoalide promotes EGFR-TKI sensitivity of lung cancer cells by KRAS-ERK pathway and mitochondrial Ca2+ overload-induced ferroptosis
Source: Front Pharmacol. 2023 Jan 11;13:1109822. doi: 10.3389/fphar.2022.1109822 (PMC9873971; doi:10.3389/fphar.2022.1109822)

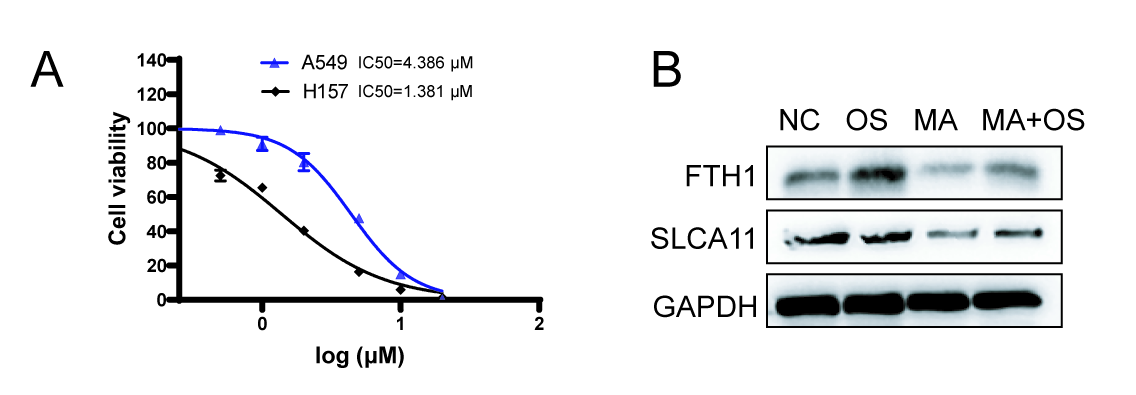

Supplement: Supplementary file 1 [file Image6.TIF]

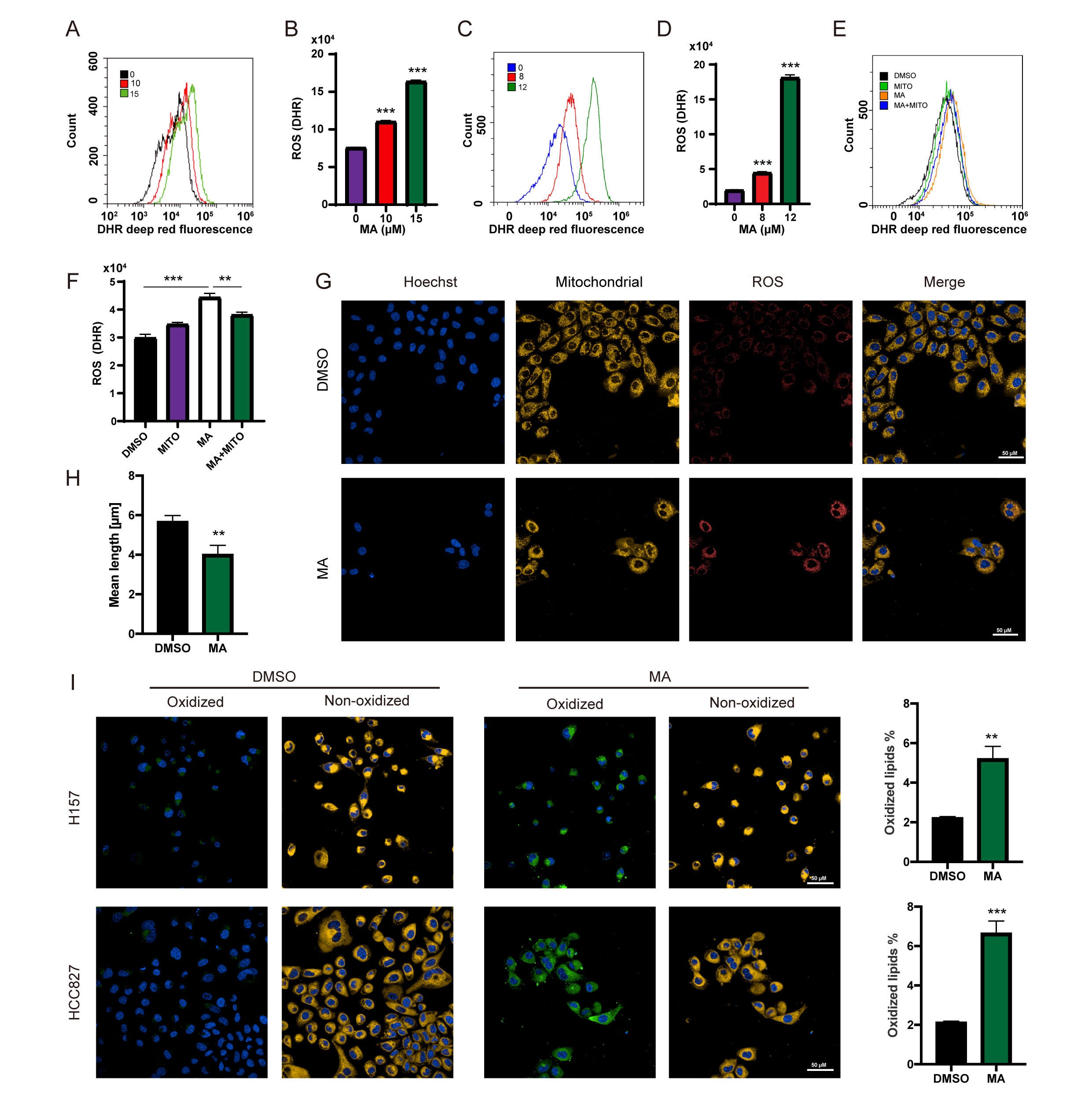

Supplement: Supplementary file 2 [file Image3.TIF]

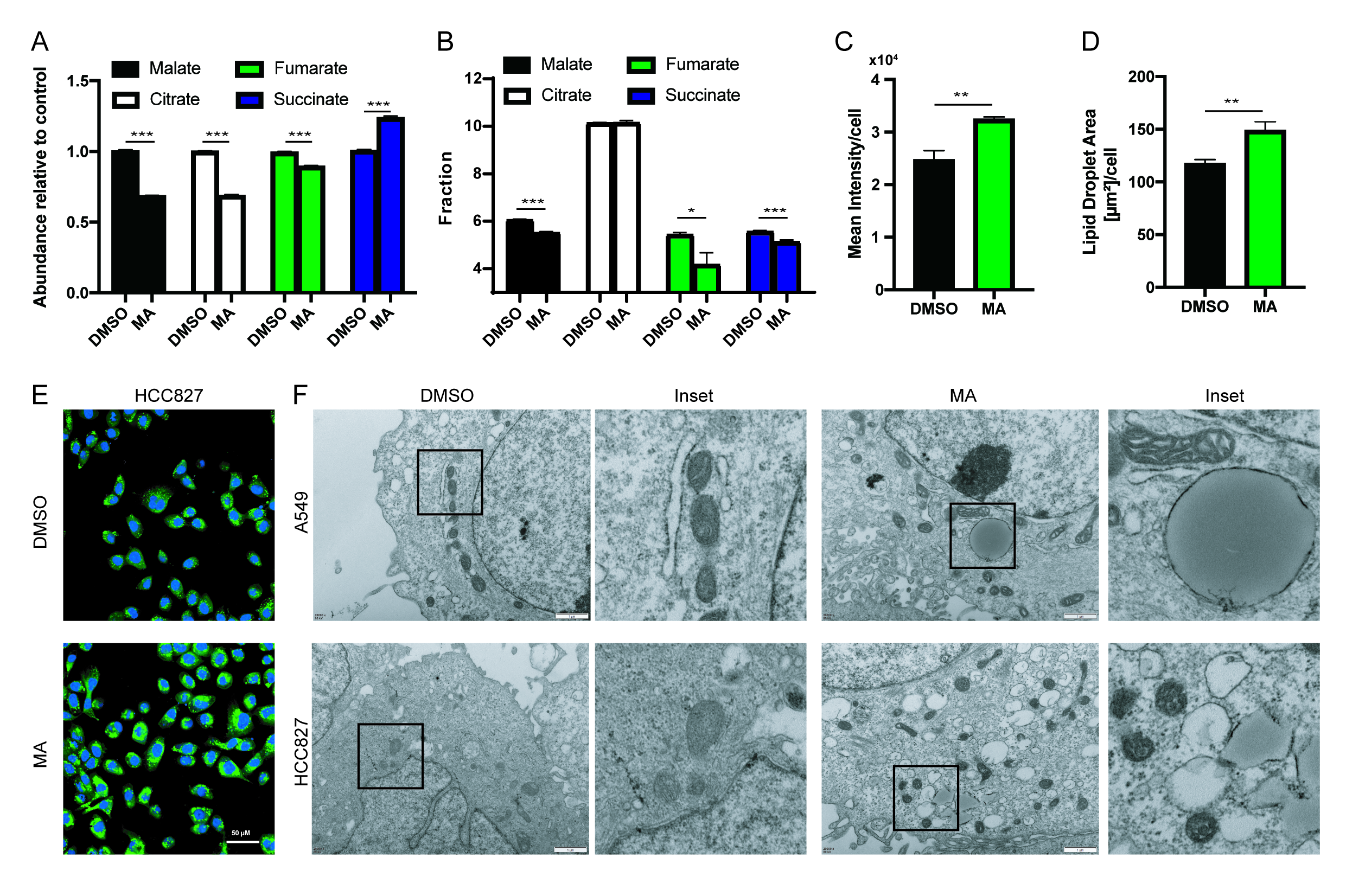

Supplement: Supplementary file 3 [file Image4.TIF]

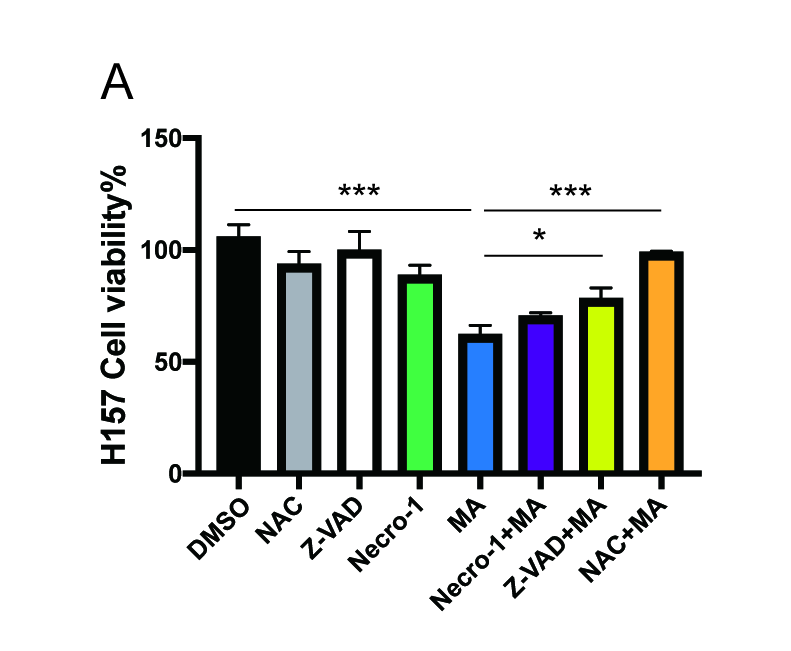

Supplement: Supplementary file 4 [file Image2.TIF]

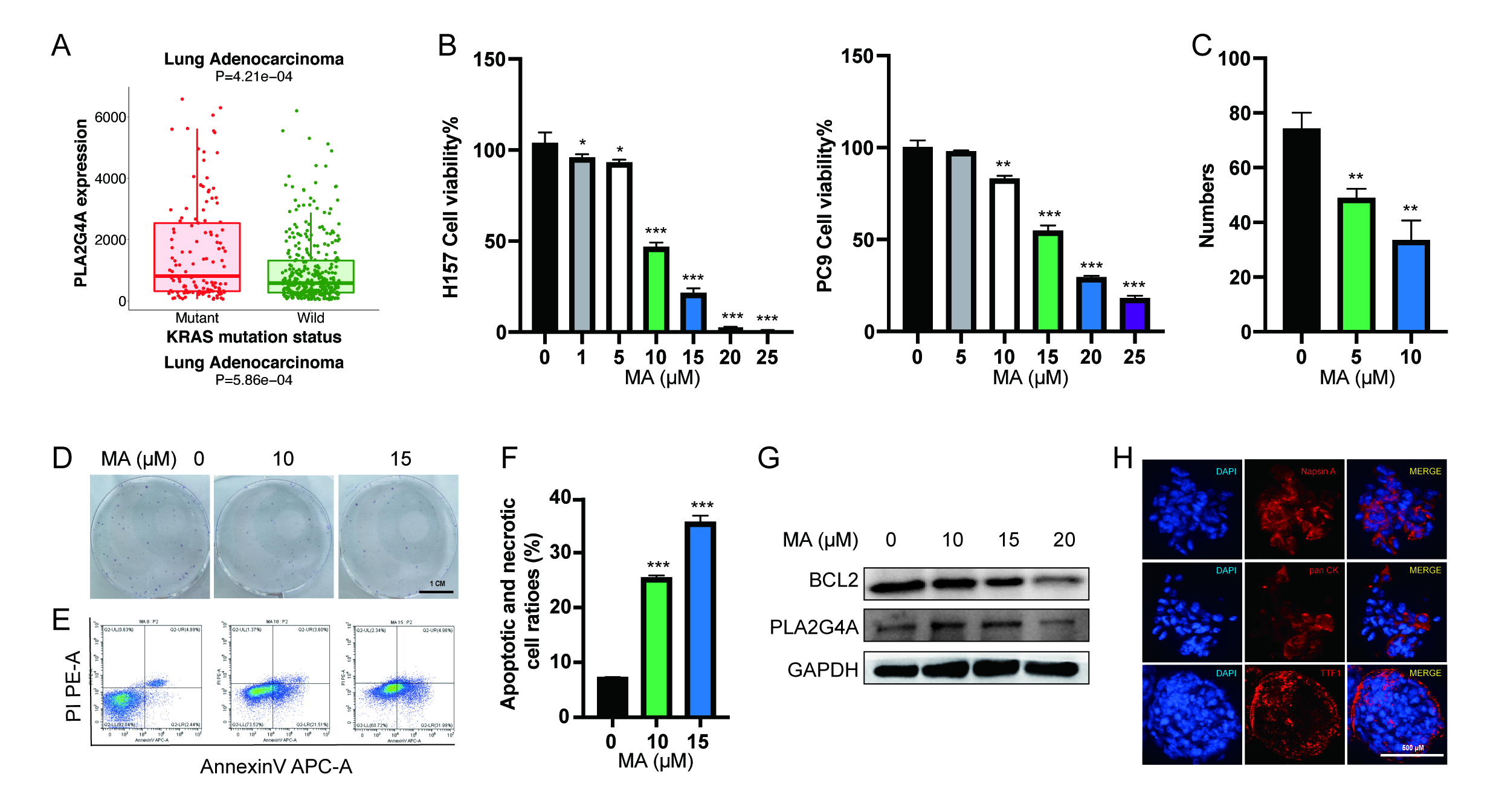

Supplement: Supplementary file 5 [file Image1.TIF]

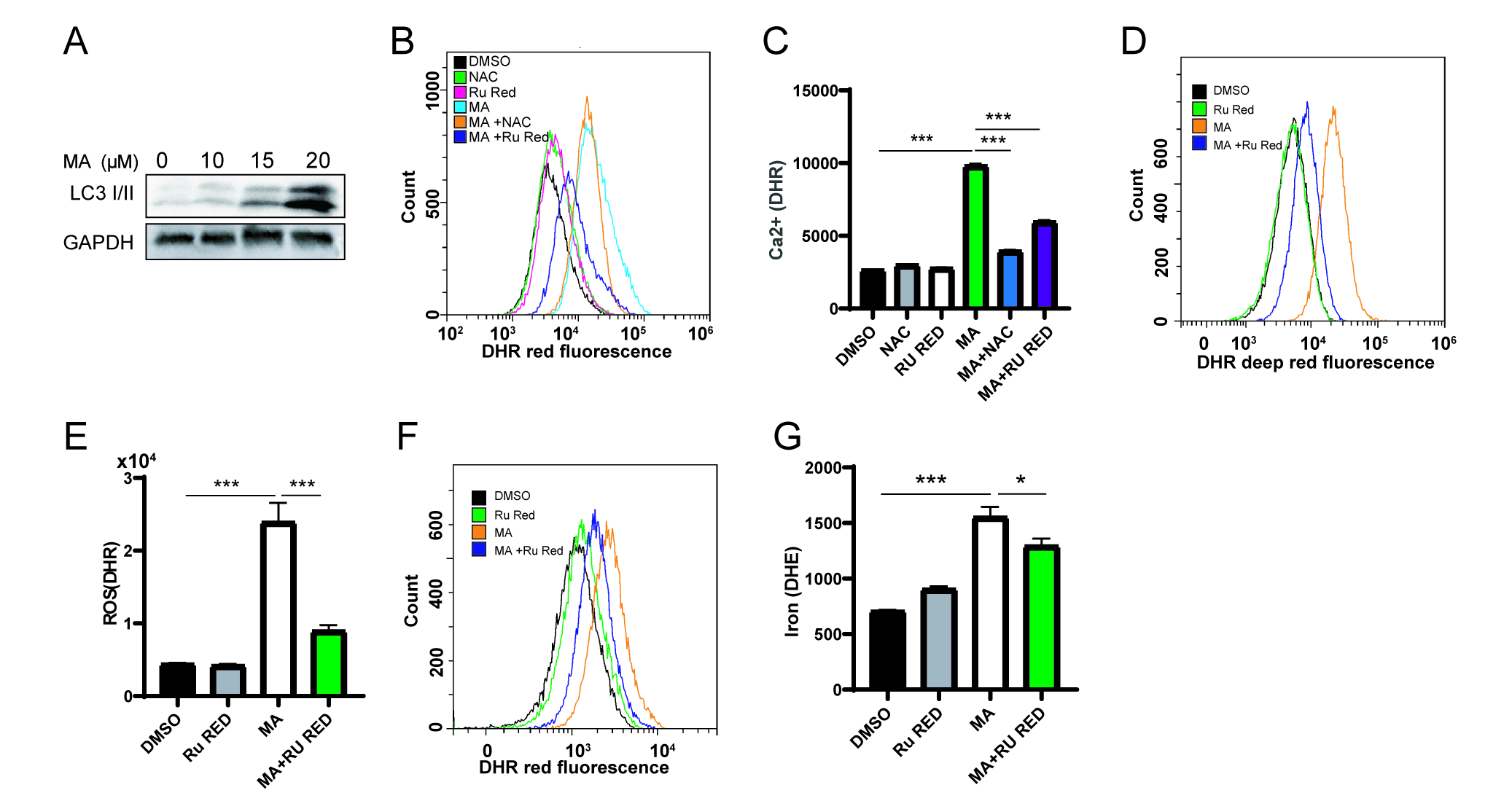

Supplement: Supplementary file 6 [file Image5.TIF]
